# Empowering coffee farming using counterfactual recommendation based RNN driven IoT integrated soil quality command system

**DOI:** 10.1038/s41598-024-56954-x

**Published:** 2024-03-15

**Authors:** Raveena Selvanarayanan, Surendran Rajendran, Sameer Algburi, Osamah Ibrahim Khalaf, Habib Hamam

**Affiliations:** 1 Department of Computer Science and Engineering, Saveetha School of Engineering, Saveetha Institute of Medical and Technical Sciences, Chennai, 602 117 India; 2 College of Engineering Techniques, Al-Kitab University, Kirkuk, Iraq; 3 Department of Solar, Al-Nahrain Research Centre for Renewable Energy, Al-Nahrain University, Jadriya, Baghdad, Iraq; 4 University de Moncton, Moncton, NB E1A 3E9 Canada

**Keywords:** Soil monitoring system, Monitoring sensor, Recurrent neural network (RNN), Gated recurrent units (GRU), IoT sensor, Counterfactual recommendation, Environmental biotechnology, Environmental impact, Computational science, Computer science, Information technology

## Abstract

Soil health is essential for whirling stale soil into rich coffee-growing land. By keeping healthy soil, coffee producers may improve plant growth, leaf health, buds, cherry and bean quality, and yield. Traditional soil monitoring is tedious, time-consuming, and error-prone. Enhancing the monitoring system using AI-based IoT technologies for quick and precise changes. Integrated soil fertility control system to optimize soil health, maximize efficiency, promote sustainability, and prevent crop threads using real-time data analysis to turn infertile land into fertile land. The RNN-IoT approach uses IoT sensors in the coffee plantation to collect real-time data on soil temperature, moisture, pH, nutrient levels, weather, CO2 levels, EC, TDS, and historical data. Data transmission using a wireless cloud platform. Testing and training using recurrent neural networks (RNNs) and gated recurrent units gathered data for predicting soil conditions and crop hazards. Researchers are carrying out detailed qualitative testing to evaluate the proposed RNN-IoT approach. Utilize counterfactual recommendations for developing alternative strategies for irrigation, fertilization, fertilizer regulation, and crop management, taking into account the existing soil conditions, forecasts, and historical data. The accuracy is evaluated by comparing it to other deep learning algorithms. The utilization of the RNN-IoT methodology for soil health monitoring enhances both efficiency and accuracy in comparison to conventional soil monitoring methods. Minimized the ecological impact by minimizing water and fertilizer utilization. Enhanced farmer decision-making and data accessibility with a mobile application that provides real-time data, AI-generated suggestions, and the ability to detect possible crop hazards for swift action.

## Introduction

In the nineteenth century, coffee cultivation in Brazil began to expand into the Cerrado region. The Cerrado is a vast region of savanna with poor soils. However, Brazilian coffee farmers developed new techniques for improving the soil, such as using lime and fertilizer. As a result, the Cerrado is now the world's largest coffee-producing region. The components nitrogen, phosphorus, potassium, calcium, magnesium, Sulphur, and iron can all be found in soil that is considered fertile^1^. The optimal soil for growing coffee is loamy soil, found in the state of north Karnataka, India which has a good combination of texture, drainage, and water retention. Coffee plantation soil requires well-drained soil to prevent waterlogging and root rot. Coffee plants have extensive root systems that extend deep into the soil to absorb nutrients and water^2^. The presence of nutrient-rich soil serves as the fundamental basis for the optimal growth and development of coffee plants, hence facilitating the production of superior-grade coffee beans. Fertility refers to the soil's ability to provide essential nutrients for plant growth, such as nitrogen, phosphorus, and potassium. Healthy soil leads to healthier coffee plants, which produce higher yields of high-quality beans. Coffee plants thrive in slightly acidic soil with a pH of 5.0–6.5.

Crop covering, composting, organic fertilizer, reduced tillage, water conservation, and shade management are ancient soil fertility strategies. Restoration of dry land to rich soil using IoT sensors to monitor and enhance coffee plantation soil health is creative and successful^3^. Soil sensors measure nitrogen, phosphorus, and potassium. Soil temperature sensors show how temperature affects plant growth and nutrient uptake. Farmers can protect coffee plants from extreme temperatures by tracking soil temperature patterns. Soil temperature sensors show how temperature affects plant growth and nutrient uptake. Analyzing soil temperature patterns protects coffee plants from extreme temperatures^4^. IoT sensors help farmers optimize irrigation, fertilization, and other soil management activities for healthier soil and higher crop yields by giving real-time soil data. Recurrent neural networks (RNNs) are a type of artificial neural network (ANN) that are well-suited for processing sequential data like IoT sensor time-series data. RNNs are useful for time series prediction, anomaly detection, and natural language processing because they can learn and understand temporal patterns and interdependencies in datasets. Recurrent neural networks (RNNs) provide the capability to analyze records of soil moisture data, enabling the prediction of forthcoming moisture levels. Conduct a comprehensive examination of soil nutrient data to predict potential nutrient deficits, hence facilitating the efficient and effective application of fertilizers by farmers. This research gap identifies characteristic patterns in soil sensor data, with a special emphasis on sudden changes in moisture and nutrient levels. Regular soil monitoring will monitor the alterations in soil condition and prompt safeguards will be implemented^5^. Develop predictive models that provide suggestions for optimal irrigation, fertilization, and other soil management practices. The limited scope of traditional soil testing often focuses on soil organic matter, microbial activity, and soil structure. However, the use of organic improvement in soil may result in delayed progress. Generalized suggestions derived from past data may not consider the particular soil characteristics, regional climate, or contemporary methods of management. The information provided Erroneous interpretation of soil test findings and suggestions might result in misguided conclusions and unproductive actions. Insufficient historical analysis, which fails to account for previous management techniques and local environmental conditions, can have an influence on evaluations of soil health status and long-term patterns. This paper is organized as follows: “Literature review” section presents a review of related work in Recurrent Neural Networks with the Internet of Things using deep learning. “The proposed model” section describes the proposed RNN-IoT approach in detail. “Results and discussion” section presents the experimental setup and results. “Conclusion” section discusses the results and compares RNN-IoT with existing methods. “Challenges and future possibilities” section concludes the paper and discusses future research directions.

## Literature review

Aarthi, R., Sivakumar, D., et al., proposed the optimal watering schedule and fertilizer application rate. IoT software platforms that can be used to develop smart soil property analysis systems such as Thing Speak, Blynk, Cayenne, Node-RED, and Azure IoT Hub. Smart soil property analysis systems can provide real-time soil conditions data, allowing farmers to respond quickly to any changes. Future work is to develop more sophisticated machine learning models to improve the accuracy of the predictions^6^. Na, A., Isaac, W., Varshney, S, et al., proposed an Internet of Things (IoT)-based system for remote monitoring of soil characteristics a system that employs sensors to collect data on soil properties, such as pH, electrical conductivity (EC), moisture, temperature, and then communicates this data to a cloud platform or another remote location. These soil qualities include electrical conductivity (EC), pH, and temperature^7^. Jain, N., Awasthi, Y et al., proposed an IoT-based soil analysis system using optical sensors and multivariate regression is a system that uses optical sensors to measure the color and reflectance of soil and then uses multivariate regression to predict soil properties, such as pH, organic matter, and nutrient content. IoT-based soil analysis systems can be made small and portable, making them ideal for field use. Future work on IoT-based soil analysis systems could focus on making IoT-based soil analysis systems more affordable and accessible to small-scale farmers^8^. Patil, P et al., proposed the implementation of IoT to determine the level of bicarbonate in the soil a system that uses sensors to measure the pH and electrical conductivity (EC) of soil and then uses this data to calculate the bicarbonate level. IoT-based bicarbonate detection systems can provide users with the data they need to make informed decisions about irrigation, fertilization, and other agricultural practices^9^. Adrian Z et al. proposed integrating soil pH measurement into an Internet of Things (IoT) application a concept that involves using sensors to measure soil pH and then transmitting this data to a cloud platform or other remote location using a wireless communication protocol. The dataset collected by IoT-based soil pH measurement systems typically includes soil pH, Temperature, Humidity, Electrical conductivity (EC), and Timestamp^10^ as illustrated in Table 1.

**Table 1:** Literature review for soil health based on different proposed models.

Author	Concept	Software	Disadvantage	Future scope
Ajit et al.^11^	Sensor-based IoT pH readers monitor liquid pH and wirelessly provide data to a cloud platform or remote location. Assess pH over time to make smart water, food, and chemical manufacturing decisions	Cayenne, Node-RED, Azure IoT Hub	The cost of the sensors and IoT hardware can be high, especially for large industrial facilities	Developing lower-cost sensors and IoT hardware
Kamelia et al.^12^	IoT-based monitoring system for humidity and soil acidity using wireless communication	Humidity, soil acidity (pH), temperature, and timestamp sensors	Cost, complexity, reliability	Developing lower-cost sensors and IoT hardware, making the systems easier to set up and maintain, improving the reliability of the sensors
Ogudo et al.^13^	Measurement and monitoring of soil moisture using cloud IoT and Android system	Soil moisture, temperature, timestamp sensors	Cost, complexity, reliability	Integrating IoT-based soil moisture monitoring systems with other agricultural technologies, such as precision agriculture and smart irrigation
Deshpande et al.^14^	IoT-based low-cost soil moisture and soil temperature monitoring system	ThingSpeak, Blynk, Cayenne, Node-RED, Azure IoT Hub	Complexity, reliability	Potential areas for future work on IoT-based soil moisture and soil temperature monitoring systems
Pechlivani et al.^15^	IoT-based agro-toolbox for soil analysis and environmental monitoring	Air temperature, barometric pressure, intensity of visible light	Cost, complexity, reliability	Developing lower-cost sensors and IoT hardware, making the systems even easier to set up and maintain
Vidhya et al.^16^	IoT-based soil content analysis	Soil moisture, pH, temperature, EC, nutrient content	Accuracy, calibration, data management	More precise and resilient sensors, more complex multivariate regression models, integrated IoT-based soil content analysis systems, and making them
Ayyasamy et al.^17^	Role of IoT in the protection of soil and plant life from acid rain disasters	Soil pH, soil moisture, temperature, humidity, wind speed, wind direction, rainfall pH	Cost, complexity, reliability of sensors and IoT hardware	IoT hardware, making the systems even easier to set up and maintain, improving the reliability of the sensors and IoT hardware, and developing even more sophisticated data analysis

IoT (Internet of Things) is a key enabling technology for smart agriculture, as it allows for the collection and analysis of data from sensors in real-time. IoT-based soil parameter measurement systems can provide real-time data on soil parameters, which allows farmers to respond quickly to changes. Future work on IoT-based soil parameter measurement systems could focus on making the systems easier to set up and maintain^18^. The materials and methods section describes how the soil is fertilized using IoT sensors and Cloud storage.

## The proposed model

The primary objective of this research study was to monitor the overall condition of the soil and to design an advanced algorithm called RNN-IoT. The present techniques for measuring soil health based on existing and past guidelines have yielded inaccurate results. The suggested approach has successfully addressed all of the aforementioned limitations and obtained a predictable outcome in transforming barren land into sustainable and rich soil suitable for coffee cultivation.

### Build an IoT sensor network to monitor soil health

- Moisture and temperature sensors, Purpose: Measure soil moisture and temperature, Version used: N95S31B outdoor NB.
- Carbon dioxide level sensors, Purpose:Measure CO2 concentration, Version used: CO2 concentration transmitter with 0–10 V, Measurement: CO2 concentration (0–10 V).
- Soil water level indication, Purpose: Indicate soil water level, Version used: Nordic nRF9160 SiP.
- GPS sensors, Purpose: Record location data, Version used: U-blox NEO-M8N.
- LDR soil color sensors, Purpose: Measure NPK color value, Version used: MNS2-9-W2-CM-020.
- Time stamps, Purpose: Record timestamps, Version used: Maxim Integrated DS3231.
- GPS units, Purpose: Record location data.

**Figure 1:**
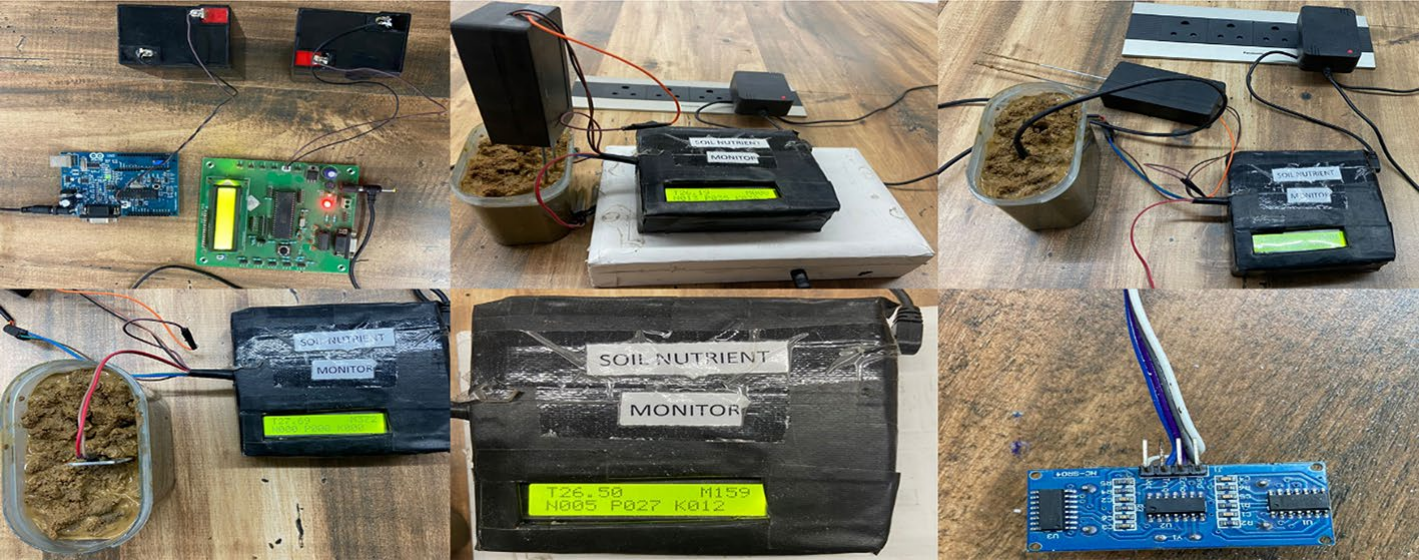
Working prototype for testing soil fertility.

Sensors measure soil potassium, phosphorus, and nitrogen levels and concentrations together with NPK color value indicators^19^. As demonstrated in Fig. 1, the N95S31B NB-IoT outdoor temperature and humidity sensor accurately measures air temperature and relative humidity.

**Figure 2:**
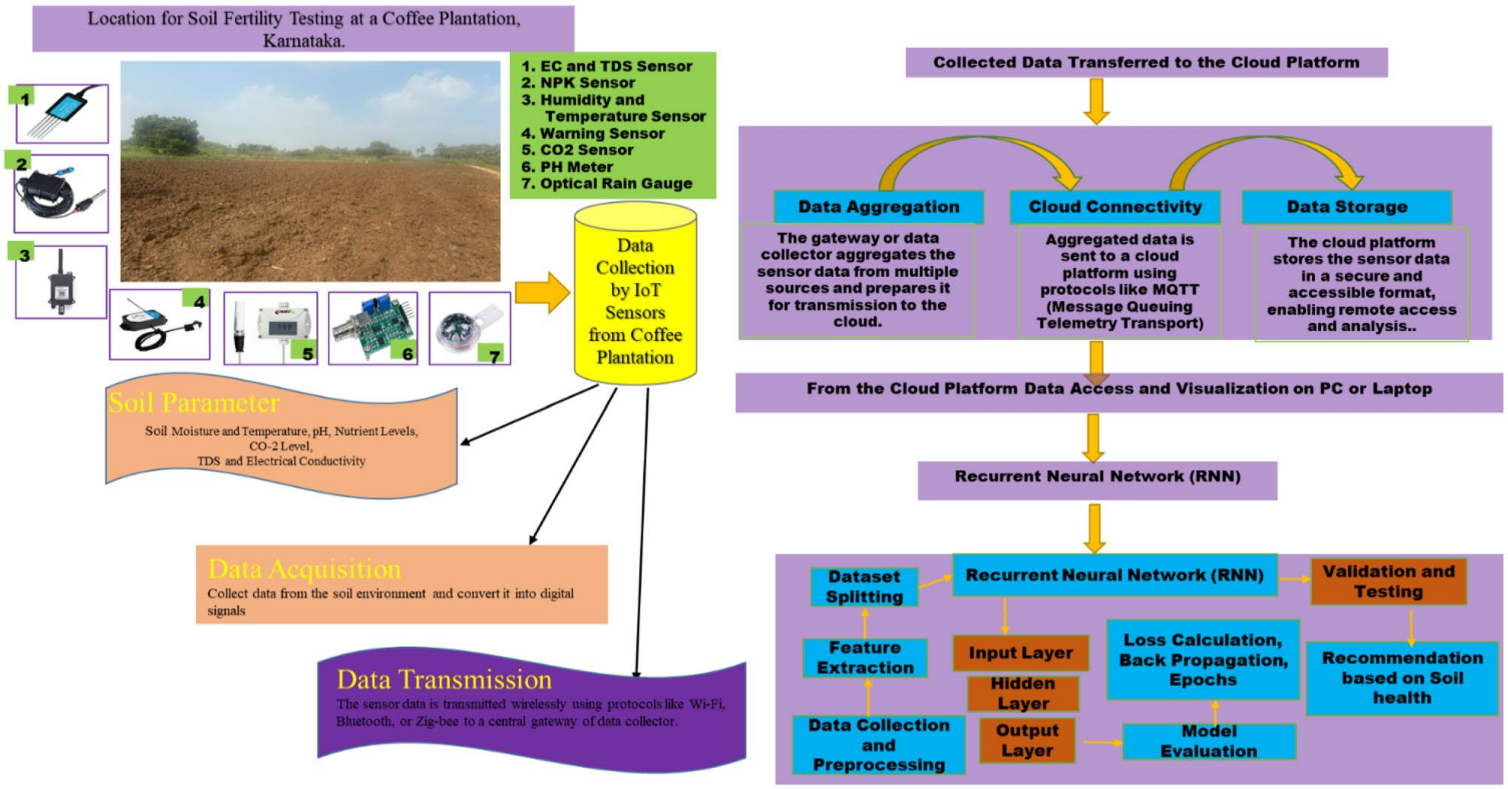
Virtual abstract for proposed system.

It subsequently sends data to an IoT server over NB-IoT. CO2 sensors measure CO. The waterproof, sturdy CO2 concentration transmitter outputs 0–10 V. Nordic nRF9160 SiP is a low-power, single-chip cellular IoT solution. Its Arm Cortex-M33 CPU, low-power RF transceiver, and multiple ports make it adaptable. The Nordic nRF9160 SiP's ADC peripheral measures soil moisture. SiP receives soil moisture sensor data^20^. The Soil EC NPK PH Sensor monitors NPK, EC, and pH. Soil electrical conductivity (EC) may indicate salt concentration. Higher EC values imply plant-harming soil salt. Growing plants need nitrogen, phosphorus, and potassium. It means 20% nitrogen, phosphorus, and potassium in the soil. Soil pH indicates acidity or alkalinity. Acidic soils have pH < 7, while alkaline soils have pH > 7. Electrical conductivity sensors detect soil solution. NPK sensors evaluate soil solution light absorption at different wavelengths. This data can track soil health trends and identify areas that need specialist soil management. LDR soil color sensors like the MNS2-9-W2-CM-020 improve coffee plantation soil health monitoring^21^. The Maxim Integrated DS3231 time stamp may improve coffee plantation soil health monitoring in numerous ways. Plantation operators can use time stamps to find patterns and trends in soil health data as indicated in Fig. 2.

### Protocol for interfacing serial technology (IST)

Soil moisture sensors measure volumetric water content. Two electrodes detect soil electrical resistance, which fluctuates with water content. Sensor output is usually a soil moisture-proportional voltage or frequency as indicated in Eq. (1). Where VWC is the volumetric water content which finds the percentage of soil volume occupied by water, can be calculated based on the weight of the wet soil minus the weight of the dry soil which divides the weight of the dry soil. εa is the apparent dielectric permittivity of the soil, εb is the dielectric permittivity of dry soil, εc is the dielectric permittivity of water. Where ρn is the neutron count rate in the soil, ρd is the neutron count rate in dry soil, and ρw is the neutron count rate in water as indicated in Eq. (2).

1 $${\text{VWC}} = \left( {\upvarepsilon {\text{a}} -\upvarepsilon {\text{b}}} \right)/\left( {\upvarepsilon {\text{w}} -\upvarepsilon {\text{c}}} \right)$$

2 $${\text{VWC}} = \left( {\uprho {\text{n}} -\uprho {\text{d}}} \right)/\left( {\uprho {\text{w}} -\uprho {\text{d}}} \right)$$

3 $${\text{VWC}} = {\text{M}} + {\text{N}}*{\text{R}}$$

where R is soil electrical resistance and M and N are calibration constants from Eq. (3). A soil temperature sensor is needed to understand soil activity and plant growth^22^. It employs thermistors or thermocouples, whose resistance changes with temperature. Equation (4) shows that the sensor's voltage or current output is proportional to soil temperature. Where T is soil temperature, R is thermistor electrical resistance, and A and B are calibration coefficients.

4 $${\text{T}} = {\text{A}} + {\text{B}}*\ln \left( {\text{R}} \right)$$

Water level indicators assess soil water table depth. It employs a pressure transducer or ultrasonic sensor to detect water-induced pressure or sound reflection. Sensor output is generally a water level-proportional voltage or frequency. An NPK color value indicator evaluates soil nitrogen, phosphorus, and potassium levels. It measures soil extract color with a colorimetric sensor. Sensor output is generally an RGB color value or a numerical NPK level value. A GPS sensor locates the soil monitoring spot. GPS satellites inform it of its latitude, longitude, and altitude. The sensor sends NMEA messages with location coordinates as indicated in Eqs. (5), (6), and (7).

5 $${\text{Latitude}}={\text{ArcTan}}2\left({\text{sin}}\left({\upvarphi }\right),{\text{cos}}\left({\upvarphi }\right)*{\text{cos}}\left(\uplambda \right)\right)$$

6 $${\text{Longitude}} = {\text{ArcTan}}2\left( {\sin \left(\uplambda \right),\cos \left(\uplambda \right)*\cos \left( {\upvarphi } \right) - \sin \left( {\upvarphi } \right)*\sin \left(\updelta \right)} \right)$$

7 $${\text{Altitude}} = {\text{R}}*\left( {\sin \left( {{\upvarphi } - {\upvarphi }_{0} } \right) - \left( {{\text{R}}_{0} + {\text{h}}} \right)/{\text{R}}*\sin \left( {{\upvarphi }_{0} } \right)} \right)$$

where ${\upvarphi }$ is the geodetic latitude, $\uplambda$ is the longitude, $\updelta$ is the sun’s declination, R is the Earth's mean radius (6371 km), R_0_ is its equatorial radius (6378 km), h is the GPS antenna’s height above the Earth’s surface, and ${\upvarphi }_{0}$ is the reference ellipsoid’s latitude (43.66° LDRs (light-dependent resistors) measure soil color to determine nutrient content. It usually uses an LDR, which changes resistance when exposed to different wavelengths of light. Sensor output is generally a resistance value that fluctuates proportionately with soil color as indicated in Eq. (8).

8 $${\text{R}}=({\text{Cr}}+{\text{Cg}}+{\text{Cb}})/({\text{Cw}}+{\text{Cg}}+{\text{Cb}})$$

where R is the reflected light, Cr is the red light component of the incident light, Cg is the green light component of the incident light, Cb is the blue light component of the incident light, and Cw is the white light component of the incident light. Soils with higher organic matter content tend to reflect more red light and less blue light, while soils with lower organic matter content tend to reflect more blue light and less red light. A pH sensor monitors the acidity or alkalinity of the soil. Typically, it employs a glass electrode that generates a voltage that varies by the pH of the solution that it is surrounded by. The output of the sensor is often in the form of a voltage, and its magnitude is typically proportional to the pH of the soil as shown in Eq. (9).

9 $${\text{pH}}=-{\text{log}}10\left({\text{aH}}+\right)$$

pH is soil acidity or alkalinity and pH+ is hydrogen ion activity in the soil solution. Each soil health measurement is timestamped. Even when the gadget is off, its real-time clock (RTC) preserves precise time. Synchronizing soil health data with other sources and tracking changes requires the timestamp. Table 2 estimates dry land-rich soil in coffee plantations^2^. IoT sensors may be connected to Arduino using IST. The simple and efficient IST protocol is ideal for connecting many sensors to an Arduino microcontroller. It includes Master Out Slave, in Master in Slave Out, Slave Select, and Serial Clock. SCLK synchronizes master–slave data transfer. MOSI and MISO signals send data from master to slave and slave to master. The owner chooses the slave via SS. A start condition from the master device starts IST communication with the slave. Most starts are high-to-low SS signal changes. After supplying the start condition, the master device can send a MOSI bit per SCLK clock pulse to the slave device. The slave device receives MISO data and delivers a bit to the master device for each SCLK clock pulse. Stop signals from master devices to slave devices halt IST communication. SS signals typically rise during a standstill. Arduino microcontroller board with enough computing power for data handling and communication interfaces. Use appropriate cables to connect each sensor to the microcontroller board for proper pinout and voltage. In Algorithm 1, each sensor's communication protocols are configured for data acquisition.

**Table 2:** Parameters to fertile the coffee plantation soil from dry land.

Parameter	Ideal value	Range	Effect on coffee plants
Humidity	60–70%	50–80%	Too low humidity—stress the plants and reduce their yields. Too high humidity—create favorable conditions for pests and diseases
Temperature	18–25 °C (64–77°F)	15–30 °C (59–86°F)	Temperatures below 15 °C (59°F) can damage the plants Temperatures above 30 °C (86°F) can reduce their yields
Moisture	Moderate	Not too wet or too dry	Too much moisture—lead to root rot and other fungal diseases. Too little moisture—stresses the plants and reduces their yields
Soil pH	6.0–7.0	5.5–7.5	Too low pH—difficult for the plants to absorb nutrients. Too high pH—reduce the availability of iron and other essential nutrients
Soil EC	0.5–2.0 ds/m	0.2–3.0 ds/m	Coffee plants are sensitive to high levels of salinity. Higher EC levels can stress the plants and reduce their yields
Nutrient value	Varies depending on the nutrient	Varies depending on the nutrient	Nutrient deficiencies can reduce the yields and quality of the coffee beans
Timestamp	Track changes in soil conditions over time	Not applicable	By tracking the timestamp of the data collected, farmers can identify trends and patterns in the soil parameters

**Algorithm 1:**
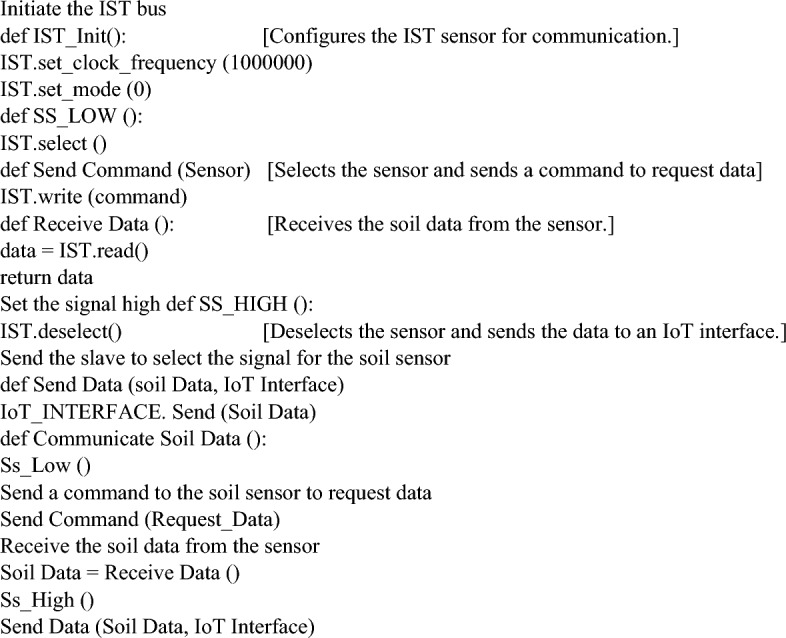
Interfacing Serial Technologies (IST)

### Configuration of sensor data transmission for arduino microcontroller

**Figure 3:**
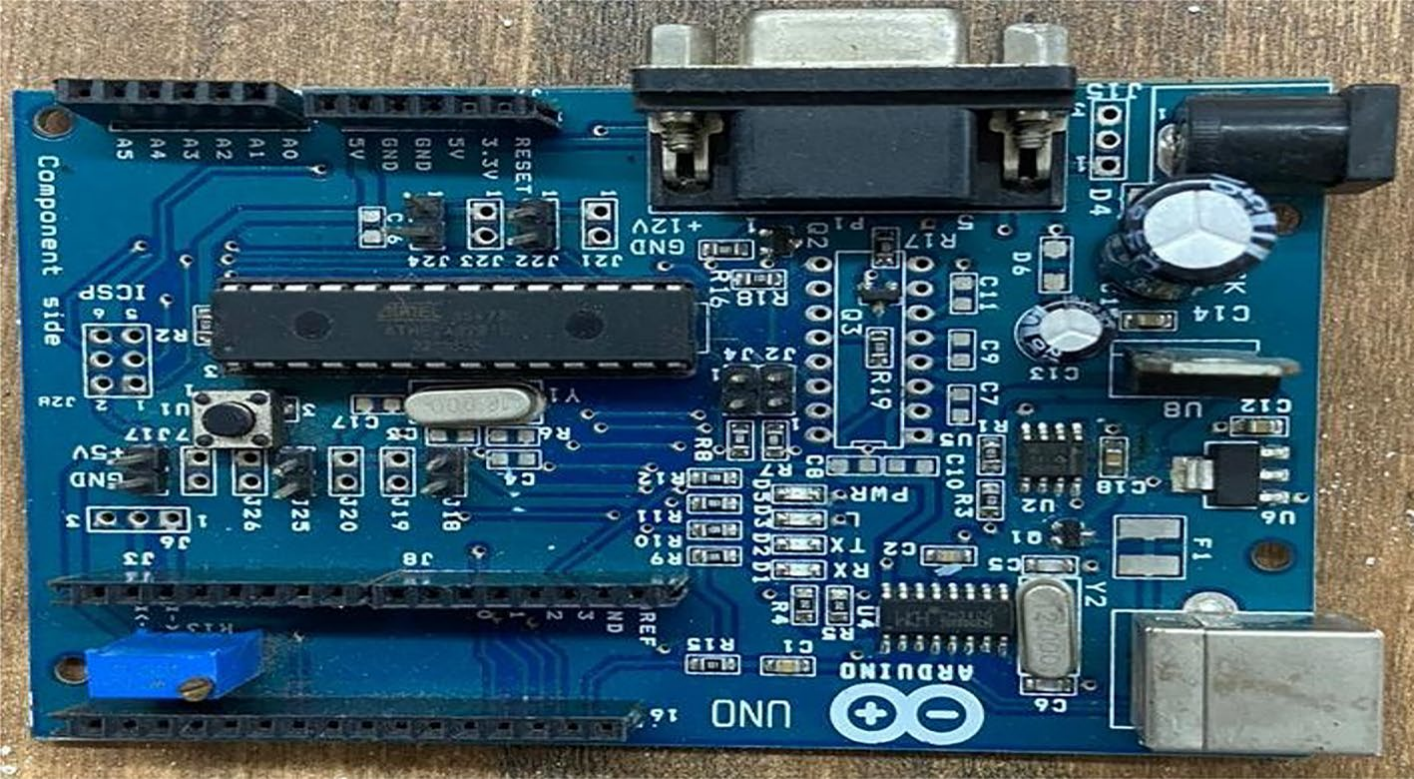
Sensors connected to an Arduino microcontroller.

For analysts to collect precise measurements of crucial soil characteristics like temperature, moisture, PH, CO2, fertilizer level, and nutrient concentrations, sensors are submerged far below the surface of the soil^23^. The Arduino microcontroller serves as the system's primary central processing unit (CPU). It is in charge of gathering information from the sensors, processing that information, and then sending it to a computer or Laptop, Mobile Phone for utilization as indicated in Fig. 3. Arduino Uno, Wi-Fi module ESP8266, Jumper wires, breadboard, and 5 V Power Supply.

### From an Arduino microcontroller to a Node MCU to the cloud

**Figure 4:**
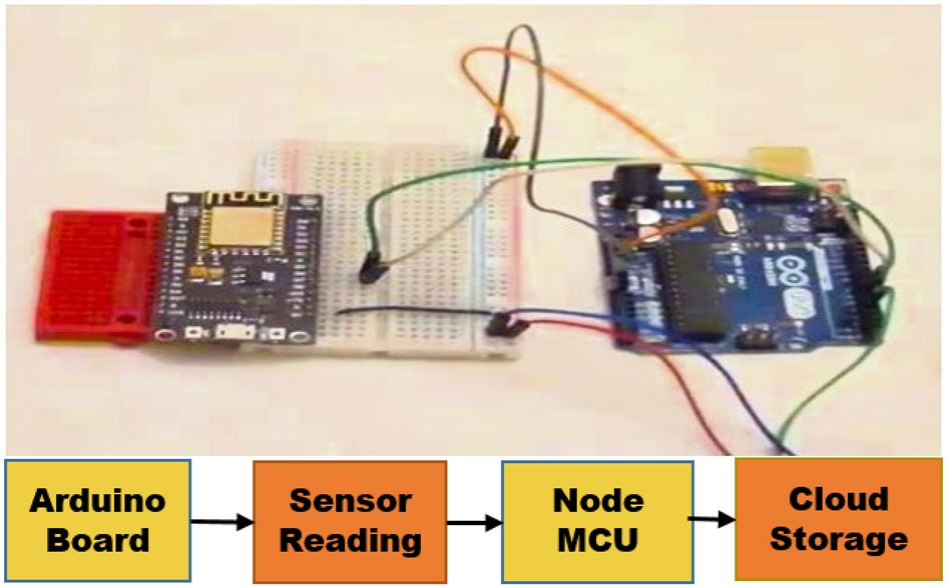
The connection between Arduino board to cloud storage.

Arduino microcontrollers capture soil health data from connected sensors as the main data acquisition unit communicating with linked sensors, reading output values, and processing sensor data as requested. Arduino microcontrollers gather soil health data from sensors as the main data-collecting unit communicating with linked sensors, reading output values, and processing sensor data^24^. The Node MCU stores data in the cloud and uses AWS IoT Core for visualization, and these services are accessed through the Node MCU as indicated in Fig. 4.

Cloud Interface provides dashboards or visualization tools to monitor the uploaded sensor data in real time or over time. The data can be analyzed to identify patterns, trends, and anomalies, providing valuable insights into the monitored environment or process. Analyzed sensor data can be used to trigger actions, such as sending alert messages based on soil health conditions.

### Recurrent neural network

The raw data was delivered to the input layer, where it was often preprocessed and normalized to ensure network interoperability. Load the CSV file into Panda’s data frame. Preprocess the data by performing necessary steps such as cleaning, handling missing values, and feature selection. Sequence formation has sequence length is a variable that determines the length of each sequence to be created. An empty list called sequences is initialized to store the generated sequences. Formula for GRU-based Soil Health Monitoring where, h_t is the hidden state of the GRU at time step t, h_{t − 1} is the hidden state of the GRU at time step t − 1, h_t̃ is the candidate hidden state at time step t, gate_update is the update gate, gate_reset is the reset gate.

10 $${h}_{t}=gat{e}_{update}*{h}_{\left\{t-1\right\}}+gat{e}_{reset}*{h}_{\widetilde{t}}$$

The update gate controls which information is allowed to flow into the hidden state from the previous time step where W_h is the weight matrix for the hidden-to-hidden state connection, U_x is the weight matrix for the input-to-hidden state connection, b_h is the bias vector for the hidden state, x_t is the input at time step t.

11 $${h}_{\widetilde{t}}=tanh\left({W}_{h}*{h}_{\left\{t-1\right\}}+{U}_{x}*{x}_{t}+{b}_{h}\right)$$

Loops from 0 to len(data)—sequence length + 1. This loop generates dataset sequences. Unfold the RNN into interconnected layers with input, forget, cell, and output gates. Input the current input into the input gate to regulate information flow into the hidden state. Apply the forget gate on the previous hidden state to determine how much to remember. Update the cell state depending on the input gate, forget gate and previous hidden state cell state that stores sequence long-term memory.

12 $${C}_{t}={f}_{t}*{C}_{\left\{t-1\right\}}+{i}_{t}*tanh\left({W}_{h}*{h}_{\left\{t-1\right\}}+{U}_{x}*{x}_{t}+{b}_{c}\right)$$

Consider C_t_ as the cell state at time step t, ${C}_{\left\{t-1\right\}}$ at time step t − 1, and ${h}_{\left\{t-1\right\}}$ as the forget gate ${i}_{t}$ is the input gate, tanh is the hyperbolic tangent activation function, ${W}_{h}$ and ${U}_{x}$ are the hidden-to-hidden and input-to-hidden weight matrices, respectively. At time step t, ${b}_{c}$ is the bias vector for the cell state, ${x}_{t}$ is the input, and ${h}_{\left\{t-1\right\}}$ is the hidden state. Use the output gate to create the current time step output that passes cell state information. Gated recurrent units (GRUs) architecture trains the dataset properly depending on soil prediction features. At each time step, calculate the error between expected and actual output. Back propagates the error via the unrolled RNN to calculate weight error gradients. Update weights to reduce inaccuracy. Prediction and Analysis in the RNN model increase warnings and suggestions by predicting soil health metrics from real-time sensor inputs. Historical sensor data, growth trends, and FFNN soil health results train the model^25^. Change specifics to simulate scenarios.

### Counter-factual recommendation generation

**Figure 5:**
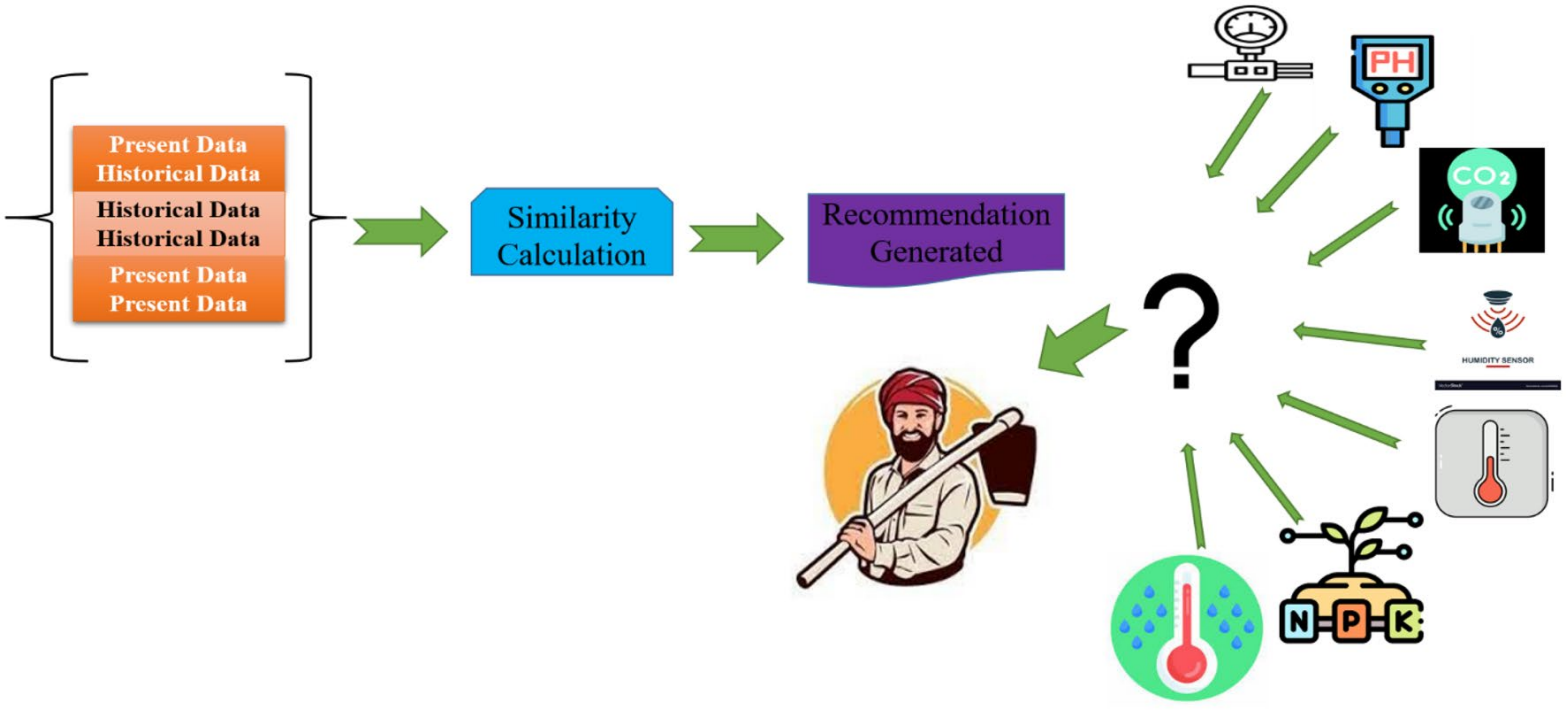
Counter-factual recommendations.

Parameters within historical data to represent various ‘what-if’ situations. It compares with the actual generated out with historical data to generate recommendations. They are comparing the recommendation using three ways Present data—Historical data, Historical data—Historical data, and Present data—Present data as illustrated in Fig. 5.

For example, let us contemplate a hypothetical situation in which a soil health monitoring system, based on feedforward neural network (FFNN) architecture, provides a recommendation for the optimal application rate of fertilizer for a particular agricultural area. Counterfactual suggestion has the potential to be employed to generate alternative fertilizer recommendations. Manhattan distance, often known as taxi cab distance, is calculated by adding the absolute coordinate differences of two places. Imagine traveling solely horizontally or vertically in Manhattan’s grid. Distance is calculated using present and historical data. Consider N points in K-dimensional space, where 2 <  = N <  = 10^ {5} and 1 <  = K <  = 5. Find the location with the lowest Manhattan distance from the N places. Manhattan distance is two points measured along right-angle axes. It is |x_1_ − x_2_| +|y_1_ − y_2_| on a plane with p_1_ at (x_1_, y_1_) and p_2_ at (x_2_, y_2_). Sorting the points in all K dimensions and outputting the middle components of each dimension reduces the Manhattan distance.

13 $$Manhattan\;Distance = \left| {s1 - s2} \right| + \left| {r1 - r2} \right|$$

The Manhattan Distance, which indicates the dissimilarity between the current suggestion and several alternative fertilizer options, may be calculated as shown in Eq. (13), where |s_1_ − s_2_| denotes the absolute difference between the two points’ s-coordinates. |r_1_ − r_2_| is the absolute difference in r-coordinates. The calculation considers nutritional content, application rates, and environmental effects. The system may create counterfactual suggestions with Manhattan Distances below a threshold. This keeps alternate options comparable to the original advice while allowing for varied perspectives. The implementation phase collects and analyzes data.

## Results and discussion

**Figure 6:**
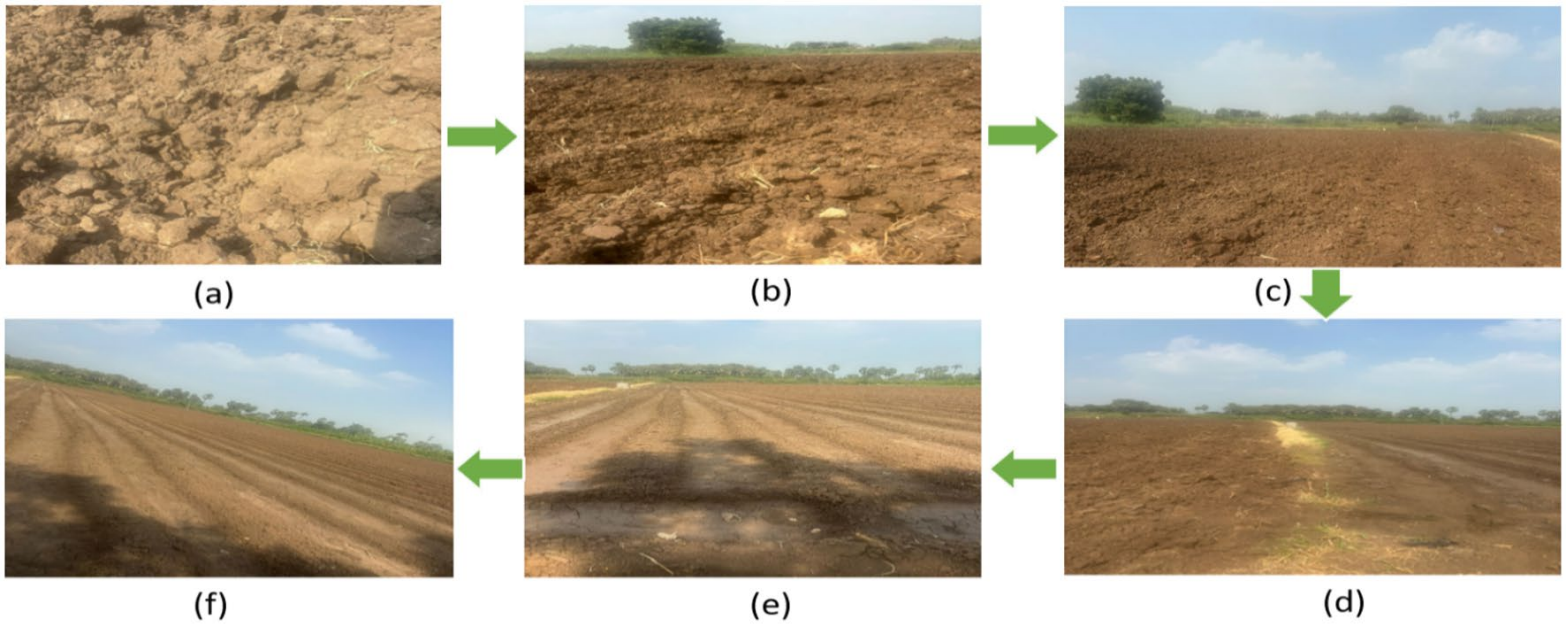
Agriculture land setup for testing soil fertility. (**a**) Agricultural infertile land, (**b**) 5-week monitoring land, (**c**) 6–10 week monitoring land, (**d**) 11–15 weeks monitoring land, (**e**) 16–20 weeks monitoring land, (**f**) Fertile soil.

The field implementation of the AI-powered system involved deploying IoT sensors in selected infertile agricultural fields to collect real-time soil data. These sensors continuously monitor soil properties such as moisture, temperature, pH, nutrient levels, and electrical conductivity. XG-booster is used for the pre-training model for predicting specific soil properties (e.g., nutrient content, and moisture levels) based on sensor data. The collected data was then fed into the AI system, which employed RNNs and GRUs to identify patterns and trends in soil health indicators as indicated in Fig. 6.

### Evaluation setup

Hardware setup has a Microcontroller—Arduino Uno heart of any IoT device, and it is responsible for collecting data from sensors, processing that data, and sending it to the cloud. Sensors- Soil moisture sensor, soil temperature sensor, pH sensor, and nutrient sensor. Wireless Communication Modules—Wi-Fi communication module, Bluetooth communication module, cellular communication module, Irrigation system actuator, fertilizer applicator actuator. AC power supply, DC power supply, battery, SD card, USB flash drive. Software setup has Windows operating system, python 3.6 Amazon Web Services (AWS)0.5, Data Visualization Tools—Matplotlib, simulates the suggested model on PC i5-8600 k, GeForce 1050Ti 4 GB, 16 GB RAM, 250 GB SSD, and 1 TB HDD. The suggested model is evaluated using False Negative, True Positive, True Negative, and False Positive metrics using the Recurrent Neural Network and Counter-Factual Recommendation Generation.

### Field setup for improving soil fertility

Enhancing the fertility of the soil is essential for achieving sustainable agriculture and achieving maximum crop yields. To evaluate and enhance the fertility of the soil throughout a range of monitoring periods, this experiment describes a field design that makes use of several land portions. Datasets are collected in real-time from Madikeri, Coorg, Karnataka total of 640, monthly monitoring based on historical data from the past three years recorded around 300582.

### Performance evaluation compared with RNN

A proposed model is compared to existing models to determine its accuracy. Table 3 shows random forest, SVM, K-means clustering, GAN, and feed-forward neural network. This performance is measured using several metrics, including accuracy, precision, recall, and F_1_ score. Accuracy is the most straightforward metric, measuring the proportion of correct predictions made by the model. Precision measures the proportion of correct positive predictions. Recall, also known as sensitivity, measures the proportion of actual positive instances that are correctly identified as positive.

**Table 3:** Performance analysis for the proposed model.

Sensor	Parameter	Measurement range	Optimal range
EC and TDS sensor	Electrical conductivity (EC)	0–4 ms/cm	0.5–1.5 ms/cm
TDS sensor	Total dissolved solids (TDS)	0–3000 ppm	500–1000 ppm
Fertilizer sensor	Nitrogen (N)	0–200 ppm	50–100 ppm
Fertilizer sensor	Phosphorus (P)	0–50 ppm	20–30 ppm
Fertilizer sensor	Potassium (K)	0–300 ppm	150–250 ppm
NPK sensor	Nitrogen (N)	0–200 ppm	60–100 ppm
NPK sensor	Phosphorus (P)	0–50 ppm	30–40 ppm
NPK sensor	Potassium (K)	0–300 ppm	200–300 ppm
Humidity sensor	Soil moisture	0–100%	40–60%
temperature sensor	Soil temperature	10–40 °C	20–25 °C
Carbon dioxide sensor	Carbon dioxide (CO2)	0–10,000 ppm	300–500 ppm
pH meter	Soil pH	4–10	5.5–6.5
Optical rain gauge	Rainfall	0–1000 mm/year	500–800 mm/year

14 $$Accuracy = (True\; Positives + True \;Negatives) / (Total\; Samples)$$

15 $$Precision = True\; Positives / (True\; Positives + False\; Positives)$$

16 $$Recall = True\; Positives / (True \;Positives + False \;Negatives)$$

17 $$F1 \;Score = 2 * (Precision * Recall) / (Precision + Recall)$$

18 $$Specificity = True \;Negatives / (True \;Negatives + False\; Positives)$$

**Figure 7:**
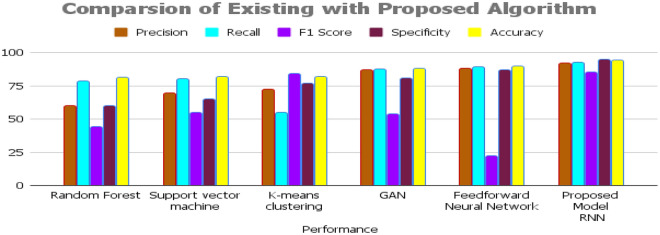
Result analysis for the proposed approach.

**Figure 8:**
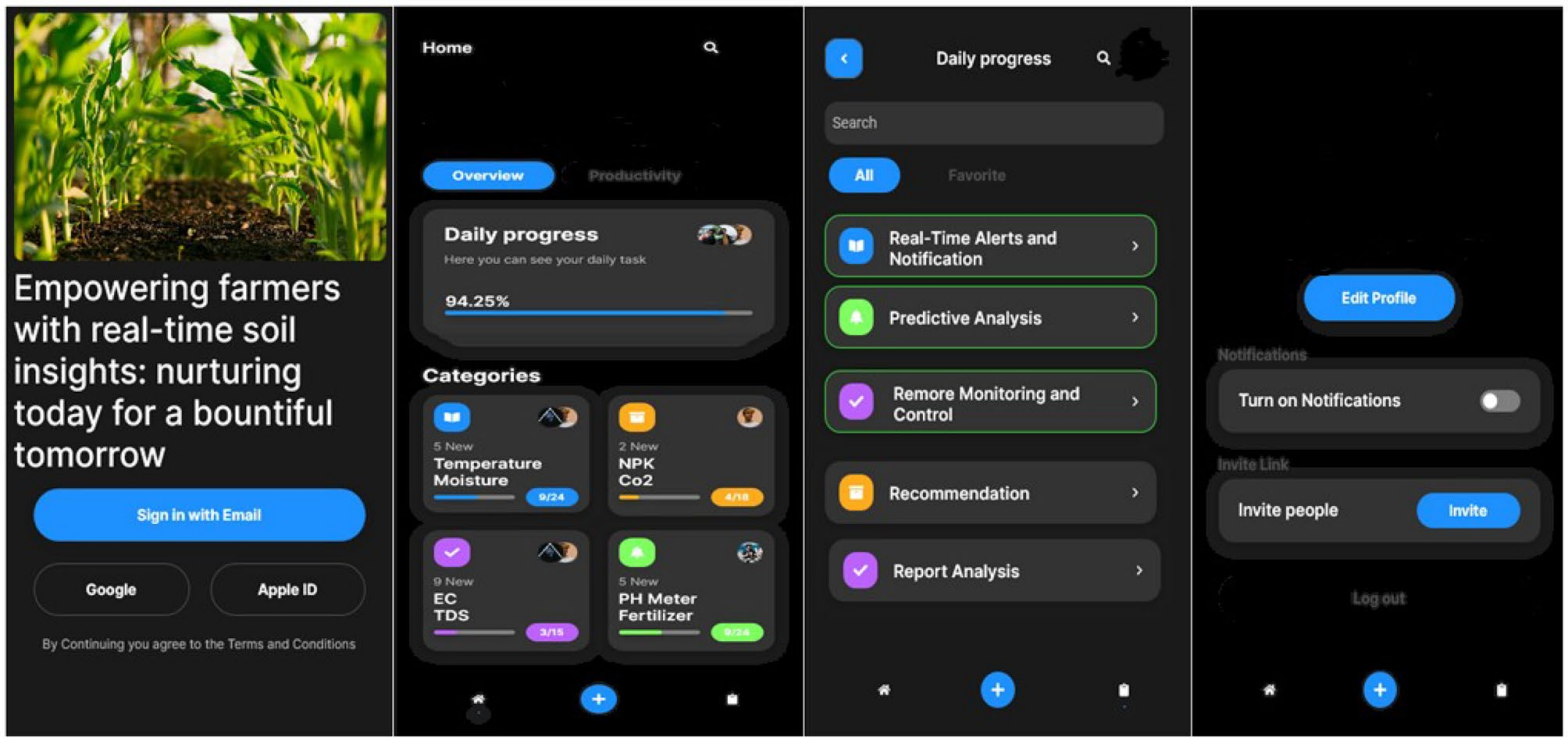
Mobile application.

The proposed method achieves 94.25% accuracy, while random forest, SVM, K-means clustering, GAN, and FFNN obtain 81.22%, 81.66%, 82.12%, 88.12%, and 89.73%. Existing approaches take longer to calculate all datasets. Figure 7 shows that the suggested technique detects events better than current methods. High EC and TDS readings indicate excessive salinity, which can stress and reduce coffee plant output. Most of the 30-day monitoring period had optimum EC and TDS values. NPK levels reflect coffee plant nutrient availability. Low nitrogen, phosphorus, and potassium (NPK) levels can decrease coffee plant output. The reduction in NPK levels from day 11–30 suggests dietary replenishment. During days 1–10, the soil moisture was a little dry, but otherwise, it was excellent. The rain prevented soil desiccation and reduced coffee plant stress. Figure 8 shows that soil temperature also affects coffee plant development. Coffee plants like soil temperatures between 20 and 25 °C. During the monitoring period, soil temperature stayed within the optimal range, promoting root growth and nutrient absorption.

### Optimization of the model

To keep the coffee plants healthy and the farm skiing, it is important to monitor the soil health of the plantations. Beans with more robust scents and tastes are a product of soil that is both healthy and well-tended. Farmers may maximize water and fertilizer efficiency and cut down on production expenses by learning what their soil requires to be healthy. To maintain healthy and fertile soil over the long term, it is important to evaluate it regularly for signs of nutrient imbalances. Weekly data analysis utilizing the RNN model is used to monitor soil conditions, and farmers are updated on health-related information and comments about soil quality. For a more precise prediction, we compare the soil condition from the second week of monitoring with that from the first week. In a similar way comparing week 2 with week 1 generates correct results, comparing week 3 with week 2 and so on also yields accurate results.

### Measurements taken to enrich the soil of coffee plantations in arid regions

When substantially monitoring and tracking various essential indicators such as electrical conductivity (EC), total dissolved solids (TDS), nitrogen (N), phosphorus (P), potassium (K), soil moisture, soil temperature, carbon dioxide (CO2), and soil pH, agricultural practitioners can promptly detect potential issues and implement appropriate measures to uphold an optimal ecosystem for their coffee crops. The soil solution's EC and TDS levels indicate salt content as indicated in Table 4.

**Table 4:** Measurement Range to fertile the coffee plantation soil from dry land.

Performance	Precision	Recall	F1 score	Specificity	Accuracy
Random forest	79.67	78.33	79.77	81.46	81.22
Support vector machine	79.69	80.23	81.56	82.56	81.66
K-means clustering	82.15	82.63	82.99	84.23	82.12
GAN	86.98	87.23	88.12	88.89	88.12
Feedforward neural network	86.97	89.23	89.91	90.12	89.73
Proposed model RNN	92.14	92.45	93.22	94.78	94.25

**Figure 9:**
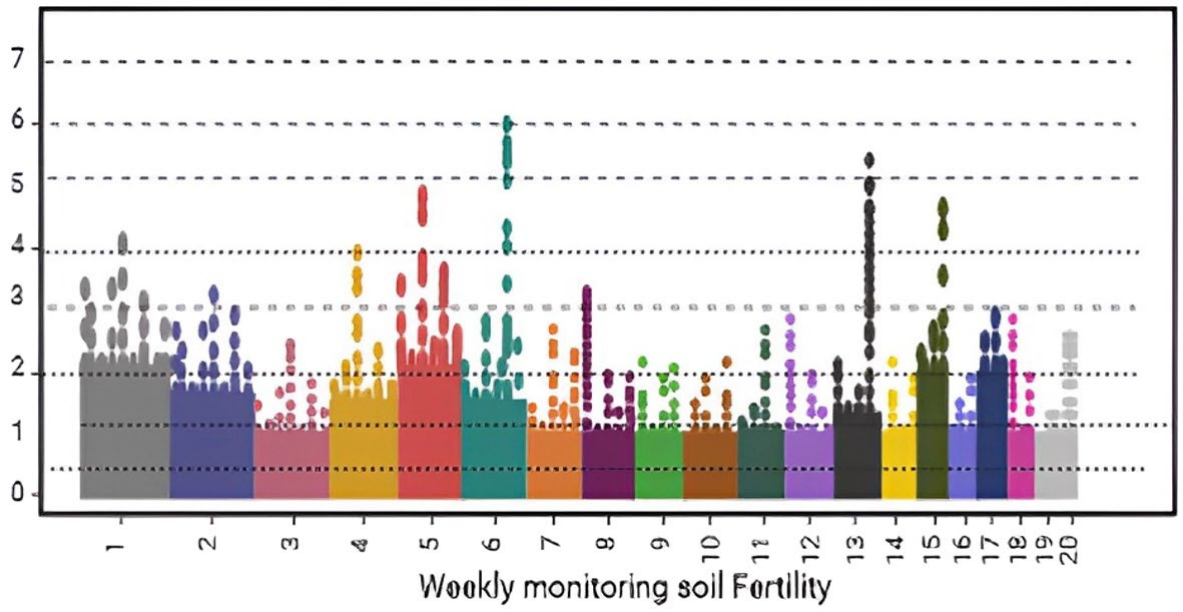
Weekly monitoring using the IoT Platform.

CO2 levels rose somewhat from day 11–20, indicating a healthier soil microbial community. Coffee plants thrive best at 5.5–6.5 soil pH as illustrated in Fig. 9. The soil pH remained within the correct range during the monitoring period, ensuring that coffee plants could easily obtain nutrients as shown in Table 5. Rainfall is vital to soil hydration. The monitoring period was without significant precipitation from day one to day fourteen. This shows that irrigation was needed to maintain soil moisture levels throughout this era. Day 15 brought 15 mm of rain, restoring soil moisture. Coffee plant soil health measurements were within optimal levels for most of the 140-day monitoring period from week 1 to week 20.

**Table 5:** Parameters to fertile the coffee plantation soil from dry land.

Weekly monitoring	EC (ms/cm)	TDS (ppm)	N (ppm)	P (ppm)	K (ppm)	Soil moisture (%)	Soil temperature (°C)	CO_2_ (ppm)	Soil pH	Rainfall (mm)
Week 1	1.2	700	60	25	180	50	22	350	6.0	10
Week 2	1.1	650	55	23	170	45	23	320	6.1	5
Week 3	1.0	600	50	20	160	40	24	300	6.2	15
Week 4	1.3	800	65	28	190	55	21	380	6.0	20
Week 5	1.2	750	60	25	180	50	22	350	6.1	10
Week 6	1.1	700	55	23	170	45	23	320	6.2	5
Week 7	1.0	650	50	20	160	40	24	300	6.3	15
Week 8	1.3	800	65	28	190	55	21	380	6.1	20
Week 9	1.2	750	60	25	180	50	22	350	6.2	10
Week 10	1.1	700	55	23	170	45	23	320	6.3	5
Week 11	1.0	650	50	20	160	40	24	300	6.4	15
Week 12	1.3	800	65	28	190	55	21	380	6.2	20
Week 13	1.2	750	60	25	180	50	22	350	6.3	10
Week 14	1.1	700	55	23	170	45	23	320	6.4	5
Week 15	1.0	650	50	20	160	40	24	300	6.5	15
Week 16	1.3	800	65	28	190	55	21	380	6.3	20
Week 17	1.2	750	60	25	180	50	22	350	6.4	10
Week 18	1.1	700	55	23	170	45	23	320	6.5	5
Week 19	1.0	650	50	20	160	40	24	300	6.6	15
Week 20	1.0	650	50	20	160	40	24	300	6.6	15

### Counter-factual recommendation

RNN-IoT model uses historical data and real-time sensor readings to predict future soil states and potential crop threats. Based on these predictions, the system generates counterfactual recommendations—alternative actions your farm could take to achieve desired outcomes.

#### Mobile application sample

The mobile application was created with the open-source Google framework Flutter, employing JavaScript. The RCNN model has been integrated and implemented, resulting in the generation of recommendations. These recommendations are created using Counterfactual suggestions.

**Figure 10:**
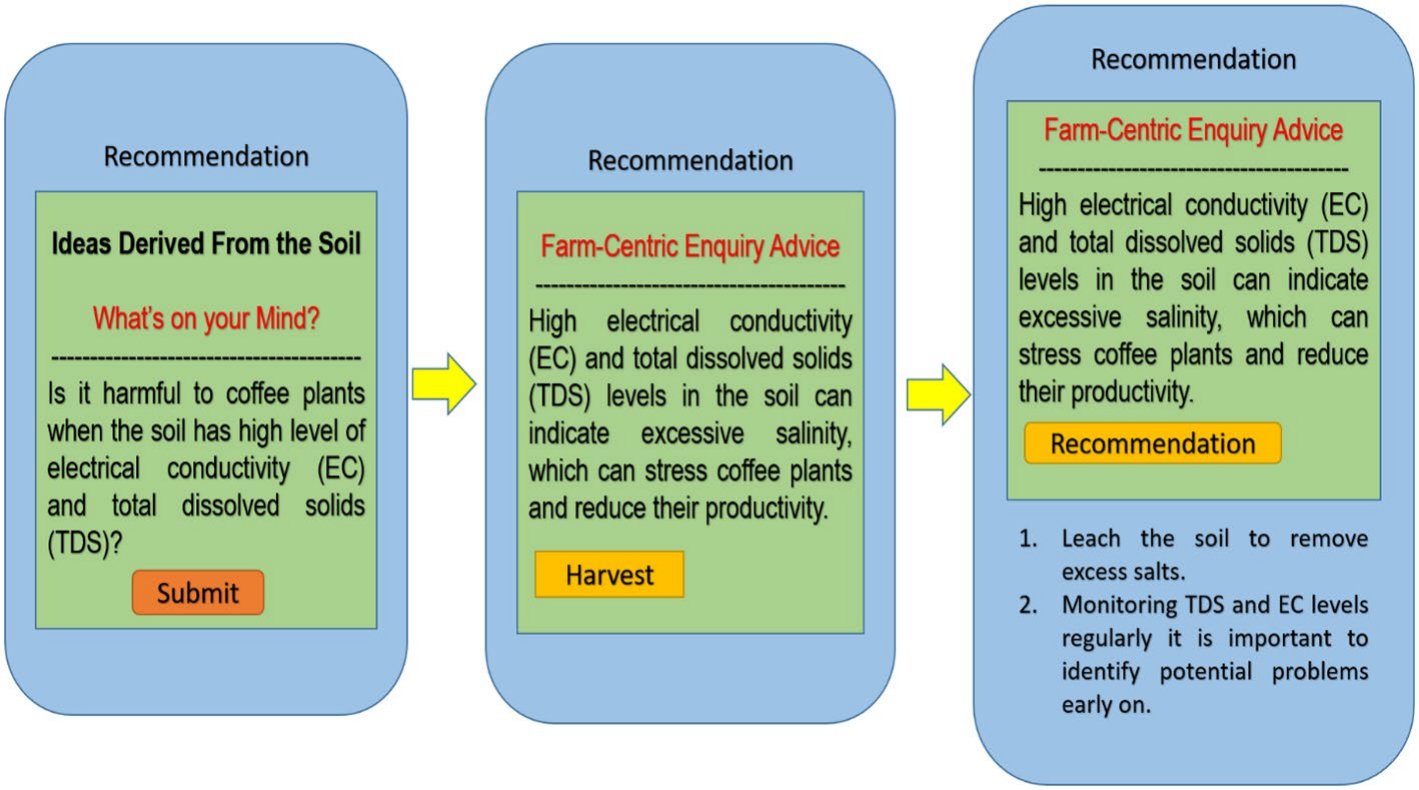
Farmer’s query-based recommendation.

**Figure 11:**
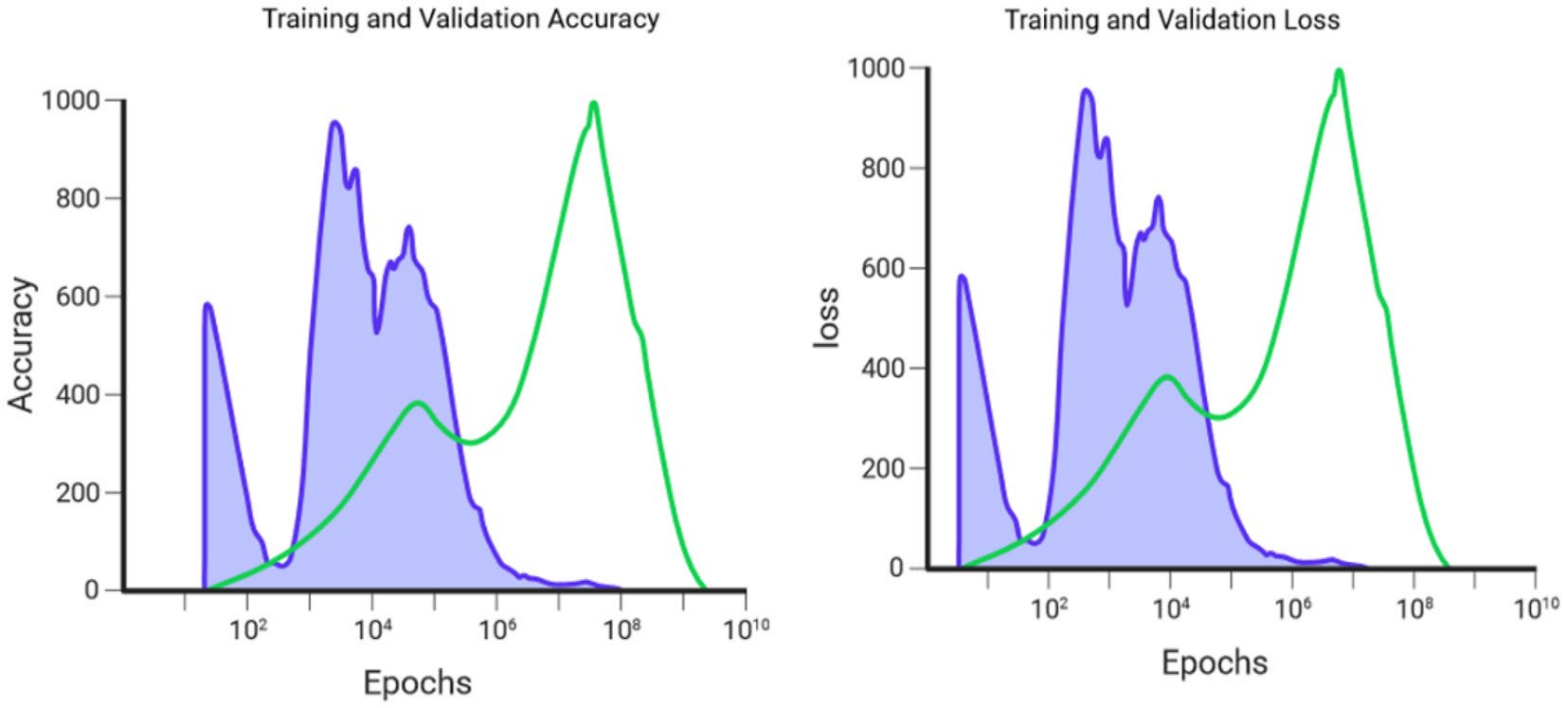
Training and validation loss versus accuracy of RNN over counterfactual recommendation.

Figure 10 is a discussion based on the question raised by the agriculturist from various parts of the coffee plantation growers. Soil-rich mobile-based applications will provide recommendations after analyzing sensor data. Agriculturist question was “How do soil conditions that are too dry, too wet, too cold, or too hot affect coffee plants”. The recommendation is generated as High or low soil pH can make it difficult for coffee plants to absorb nutrients. Solution: Apply soil amendments to adjust the pH to the optimal range. The specific type of soil amendment to apply will depend on the current soil pH and the desired pH range. Similarly, “How does rainfall affect coffee plants, and what can coffee growers do to mitigate the negative effects of extreme rainfall events”. The recommendation is generated as Insufficient or excessive rainfall can lead to water stress or soil erosion. Irrigate during periods of low rainfall and implement erosion control measures during periods of high rainfall. Monitoring rainfall patterns is important to ensure that coffee plants receive the right amount of water^26^. The proposed model is trained using the Training Set, which enables it to discover hyperlinks and patterns in the data. To minimize loss and enhance predictions. To evaluate the model's performance and adjust its hyper-parameters, a validation set is utilized during its development. Evaluates the model's ability to be extrapolated to new data, which helps avoid excess fitting. The testing set is utilized for the final and unbiased evaluation of the model's performance once all training and hyper-parameter adjustments have been finished. Ensures the model's ability to apply knowledge to unfamiliar data and offers an accurate evaluation of its capabilities as shown in Fig. 11. The total dataset is divided into 60% for training, 20% for validation, and 20% for testing. The difference between predicted and actual values is computed using a loss function by mean square error. The loss is propagated backward through time, updating model parameters (weights and biases) to minimize the loss.

**Figure 12:**
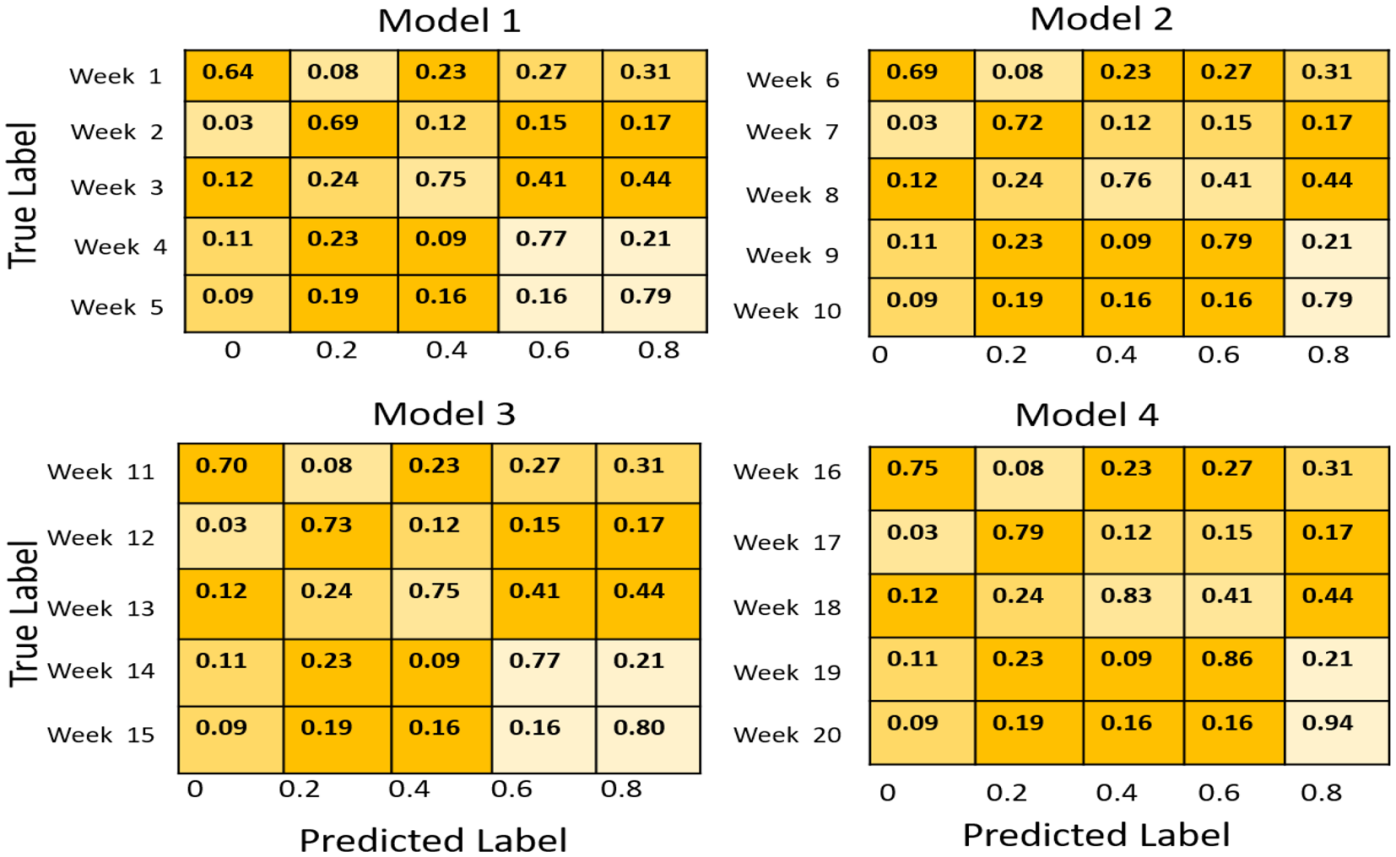
Multiple confusion matrix for monitoring soil accuracy.

A confusion matrix is an illustrative format that exhibits the success of a proposed theory^27^. The data represents the observed vs projected results for each week of monitoring. Within the realm of soil fertility, weekdays may be classified as several tiers of fertility. For instance, weeks 1–7 exhibit low fertility, weeks 8–14 demonstrate medium fertility, and weeks 15–20 showcase high fertility, making them suitable for cultivation as shown in Fig. 12.

**Figure 13:**
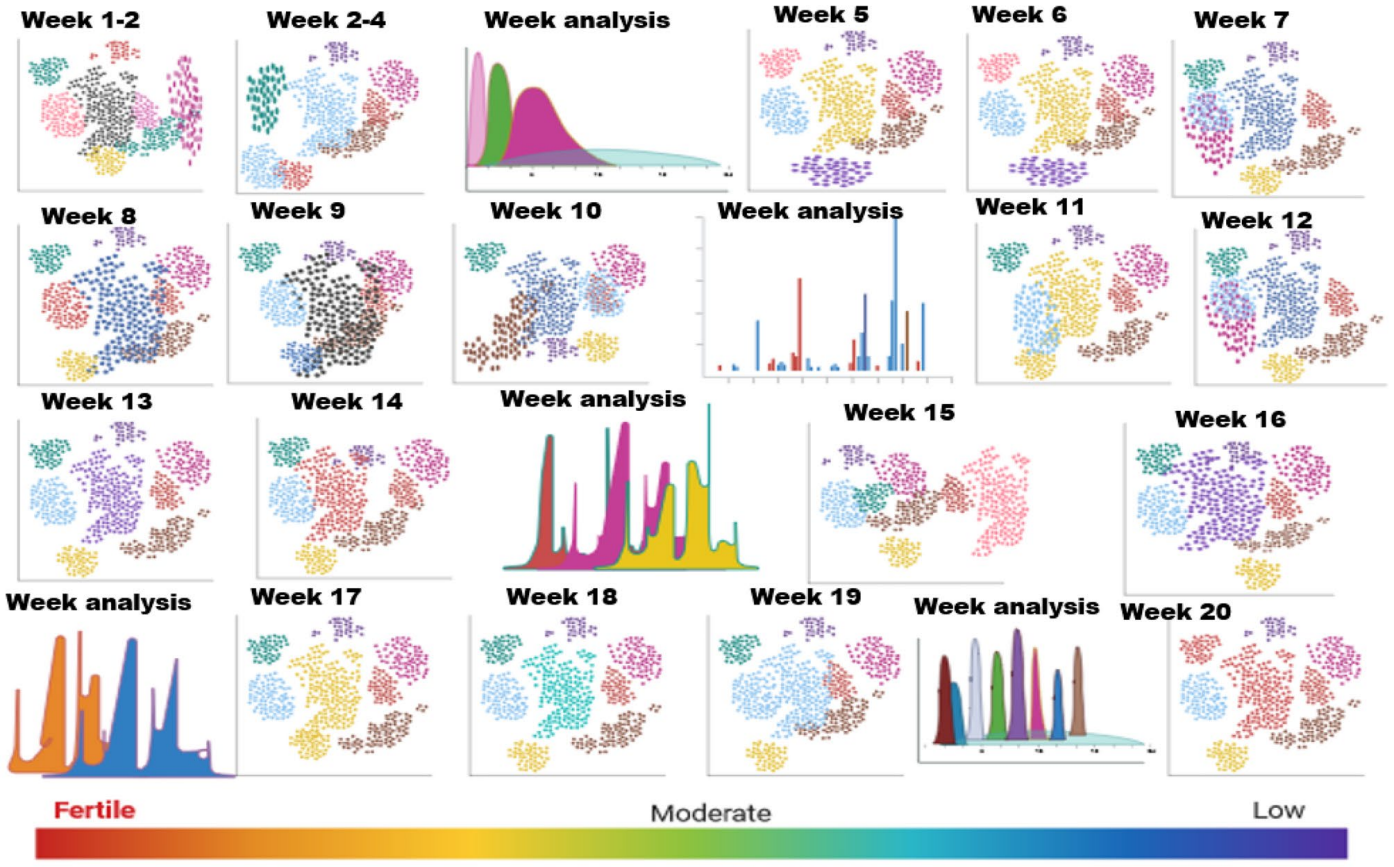
IoT sensors monitoring land view.

By using IoT sensors to track soil moisture, temperature, pH, and nutrient levels, coffee farmers can gain valuable insights into their crops' health and needs. Figure 13 data can then be used to make informed decisions about irrigation, fertilization, and other agricultural practices—a 20-week soil monitoring study that tracks the soil condition in a coffee plantation. The study was conducted in a coffee plantation in Madekari, Coorg, and Karnataka, India. Ten IoT sensors were installed on the plantation, and data was collected every hour for 20 weeks. The sensors measured soil moisture, temperature, and pH at a depth of 10 cm throughout 20 weeks. Due to heavy rainfall in the early weeks of the research, soil moisture was maximum. Dry weather diminished soil moisture. The study's soil temperature peaked in the first weeks and then dropped as the weather cooled. pH was almost consistent throughout the investigation.

20-week. The investigation produced soil moisture, temperature, and pH maps. These maps helped identify under and over-watered crop regions and regulate the irrigation system. The maps were also utilized to identify acidic and alkaline soil on the farm and adapt the fertilization program.

## Conclusion

The foreseeable future of coffee production demands beyond merely cultivating a diverse range of coffee beans; it involves establishing a sustainable and lucrative coffee business that will endure for future generations. Utilizing sophisticated sensors allows for the monitoring of environmental variables, including precipitation, humidity, and temperature, to optimize agricultural productivity. Internet of Things (IoT) devices have the potential to accurately assess the levels of water and nitrogen present in soil. Furthermore, by assessing the CO2 concentration in agricultural areas, it is possible to accurately monitor evapotranspiration rates, therefore enhancing the monitoring of soil health. This requires equipping coffee growers with the necessary tools and expertise to develop intelligent soil—a flexible and robust ecosystem that supports the growth of healthy coffee plants while reducing environmental harm. Farmers are provided with immediate updates on the condition of their soil and receive alerts on any potential issues. This enables farmers to promptly address any early signs of soil degradation. The Recurrent Neural Network and Counterfactual Recommendations utilize advanced algorithms to forecast future trends and provide tailored soil cures for youthful agriculturists. Knowledgeable farmers make well-informed choices regarding their soil, leading to higher crop production, better quality, and more profitability. The proposed method has overcome all of the above drawbacks and achieved the predictable result for converting infertile land to fertile land sustainable for coffee plantations.

The future direction is moving towards installed outside Internet of Things devices are subject to severe weather, dust, wind, temperature, and other environmental hazards. This is one of the main issues with these devices. Unexpected mechanical failure of the complex devices could occur as a result of unfavorable environmental circumstances. Therefore, the raw materials used to construct the Internet of Things (IoT) devices used in smart farming must be able to endure such harsh climatic conditions. This will ensure that the devices last longer and provide more consistent results. Coffee cherry early detection and recommendation based on advanced deep learning technology which empowers farmers to cultivate a sustainable future for coffee, one smart earth revolution at a time.

## Challenges and future possibilities

Global research on the Internet of Things (IoT) and sensor-based smart farming have revealed positive results. It is employed in several small-scale agricultural domains a rural farm, where farmers have less technological expertise, may provide more challenges. Even so, the execution of the project on a broad scale is still awaiting completion. An obstacle of great importance is the financial burden associated with the deployment and installation of IoT-tagged sensors and accessories in vast agricultural areas. Furthermore, there is ambiguity regarding the expenses associated with implementation and the monetary benefits that may be obtained. Deploying IoT-enabled technology incurs substantial expenses for hardware, software, and system operation. Supplementary costs may encompass energy usage, system upkeep, service enrollment, and labor fees for operating combined hardware and software. Enhancing farmers' digital literacy on a global scale is crucial to facilitating the extensive use of IoT technologies. Lack of comprehension and consciousness of IoT-based technologies in agriculture might result in the underutilization of intelligent systems in farming^28^. Government policymakers should develop economic strategies to facilitate the successful and efficient implementation of Internet of Things (IoT) technology by farmers in agricultural areas. Data privacy and security issues might hinder large-scale IoT and smart system adoption. Attackers can manipulate cloud server data to harm automated agricultural activities in farmlands. Attacks can negatively impact agricultural output and prevent good environmental management. IoT data security concerns contribute to the sluggish adoption of smart farming systems. Encryption is crucial for protecting critical data and digital systems in smart farming from global cyberattacks. Integrating cryptography with strong keys can reduce cyber threats on cloud systems. Additional methods, such as integrating multiparty computing with homomorphic encryption or block-chain, can yield accurate results.

### Experimental statement

No plants and Animals are disturbed for research work. All image datasets information regarding plant-based processes or applications was obtained from publicly available and ethically sourced data and publications. Plant Collection materials are complying with institutional, national, and international guidelines and legislation. So we confirm that all methods were performed in accordance with the relevant guidelines/regulations/legislation.

## Data Availability

The dataset utilized and analyzed in our research is publicly accessible to the public fertility land in the Zenodo communities Raveena. (2023). Empowering Coffee Farming Using Counterfactual Recommendation based RNN-IoT Integrated Soil Fertility Control System [Data set]. Zenodo. 10.5281/zenodo.10416960. The coding system along with additional data are accessible upon adequate request from the initial and coauthor authors.

## References

[1] Alharbi M, Rajagopal SK, Rajendran S, Alshahrani M (2023). Plant disease classification based on ConvLSTM U-net with fully connected convolutional layers. Traitement Signal.

[2] Kumar RS, Thanarajan T, Alotaibi Y (2023). Brain tumor: Hybrid feature extraction based on UNet and 3DCNN. Comput. Syst. Sci. Eng..

[3] Xiao L, Wu J, Li W, Yuan G, Xu Q, Wei J, Han F (2023). Mineral coating enhances the carbon sequestration capacity of biochar derived from *Paulownia* biowaste. Agronomy.

[4] Mustafa A, Xu H, Sun N, Liu K, Huang Q, Nezhad MTK, Xu M (2023). Long-term fertilization alters the storage and stability of soil organic carbon in Chinese paddy soil. Agronomy.

[5] Saikia D, Khatoon R (2022). Smart monitoring of soil parameters based on IoT. Int. J. Adv. Technol. Eng. Explor..

[6] Aarthi R, Sivakumar D, Mariappan V (2023). Smart soil property analysis using IoT: A case study implementation in backyard gardening. Procedia Comput. Sci..

[7] Na, A., Isaac, W., Varshney, S. & Khan, E. An IoT-based system for remote monitoring of soil characteristics. in *2016 International Conference on Information Technology (InCITe)-the next generation IT summit on the Internet of things: Connect your Worlds*, 316–320 (2016).

[8] Jain N, Awasthi Y, Jain RK (2023). An IoT-based soil analysis system using optical sensors and multivariate regression. Int. J. Exp. Res. Rev. (IJERR)..

[9] Patil, P., Vimala, M. S., Valarmathi, K. & Rose, L. Implementation of IoT to determine the level of bicarbonate in soil. in *Implementation of IoT to Determine the Level of Bicarbonate in Soil*, vol. 12, 5862–5876 (2023).

[10] Zarnescu A, Ungurelu R, Macovei MI, Varzaru G (2018). Integrating soil pH measurement into an Internet of Things application. Sci. Pap.-Ser. B Hortic..

[11] Ajit P, Gosavi, Suraj AP, Akshata G, Parulekar, Rohit S, Patade, Prashant L, Raorane (2021). IoT based pH reader. Int. J. Adv. Res. Sci. Commun. Technol. (IJARSCT).

[12] Kamelia, L., Nugraha, Y. S., Effendi, M. R. & Priatna, T. The IoT-based monitoring systems for humidity and soil acidity using wireless communication. in *2019 IEEE 5th International Conference on Wireless and Telematics (ICWT)*, 1–4 (2019).

[13] Ogudo KA, Surendran R, Khalaf OI (2023). Optimal artificial intelligence based automated skin lesion detection and classification model. Comput. Syst. Sci. Eng..

[14] Deshpande, G., Goswami, M., Kolhe, J., Khandagale, V., Khope, D., Patel, G., Doijad, R., Mujumdar, M., Singh, B. B. & Ganeshi, N. *IoT-Based Low-Cost Soil Moisture and Soil Temperature Monitoring System*, p. 23. arXiv:2206.07488 (2023).

[15] Pechlivani EM, Papadimitriou A, Pemas S, Ntinas G, Tzovaras D (2023). IoT-based agro-toolbox for soil analysis and environmental monitoring. Micromachines.

[16] Vidhya, P., Ninshiya Mary, J., Yamuna Mary, J., Suriya Ponselvi, R., & Mr, K.S. IoT-based soil content analysis. *J. Pharm. Negat. Results*. 100–112 (2023).

[17] Ayyasamy S, Jhosiah Felips JF (2023). Role of Internet of Things (IoT) in the protection of soil and plant life from acid rain disasters: A survey. IJCRT..

[18] Mutyalamma AV, Yoshitha G, Dakshyani A, Padmavathi BV (2020). Smart agriculture to measure humidity temperature moisture Ph. and nutrient values of the soil using IoT. Int. J. Eng. Adv. Technol. (IJEAT)..

[19] Subahi AF, Khalaf OI, Alotaibi Y (2022). I modified the self-adaptive Bayesian algorithm for smart heart disease prediction in IoT system. Sustainability.

[20] Raveena, S. & Surendran, R. Sustainable fertilizers in coffee plantation: Hybrid Recommendation for agricultural producers. in *2023 5th International Conference on Inventive Research in Computing Applications (ICIRCA)*, 1664–1671 (2023).

[21] Sowmiya E, Sivaranjani S (2017). Smart system monitoring on soil using the Internet of Things (IoT). Int. Res J. Eng. Technol. (IRJET).

[22] Manivasan, V., Rathinavel, J. P., Khanna, A. K. & Visu, P. Soil and water compatibility testing based on IOT. *Int. J. Adv. Netw. Appl.* 332–334 (2019).

[23] Schirrmann M, Gebbers R, Kramer E, Seidel J (2011). Soil pH mapping with an on-the-go sensor. Sensors..

[24] Raut, S. & Chitre, V. Soil monitoring and testing using IoT for fertility level and crop prediction. in *Proceedings of the 3rd International Conference on Advances in Science & Technology (ICAST)*, vol. 1, 1–19 (2020).

[25] Selvanarayanan R, Rajandran S, Alotaibi Y (2023). using hierarchical agglomerative clustering in E-nose for coffee aroma profiling: Identification, quantification, and disease detection. Instrum. Mes. Métrol..

[26] Surendran, R., Khalaf, O. I. & Tavera Romero, C. A. Deep learning based intelligent industrial fault diagnosis model. *Comput. Mater. Contin.* **70**(3) (2022).

[27] Rajak P, Ganguly A, Adhikary S, Bhattacharya S (2023). Internet of Things and smart sensors in agriculture: Scopes and challenges. J. Agric. Food Res..

[28] Tamilvizhi T, Alotaibi Y, Rajendran S (2023). It improved wolf swarm optimization with deep-learning-based movement analysis and self-regulated human activity recognition. AIMS Math..

